# i*-*Rheo-optical assay: Measuring the viscoelastic properties of multicellular spheroids

**DOI:** 10.1016/j.mtbio.2024.101066

**Published:** 2024-04-20

**Authors:** Rosalia Ferraro, Stefano Guido, Sergio Caserta, Manlio Tassieri

**Affiliations:** a DICMaPI, Università di Napoli Federico II, P.le V. Tecchio 80, 80125, Napoli, Italy; b CEINGE Advanced Biotechnologies, Via Gaetano Salvatore, 486, 80131, Napoli, Italy; c Division of Biomedical Engineering, James Watt School of Engineering, Advanced Research Centre, University of Glasgow, Glasgow, G11 6EW, UK

**Keywords:** Multicellular spheroids, Rheology, Mechanobiology, Cancer research, Optical assay

## Abstract

This study introduces a novel mechanobiology assay, named “i-Rheo-optical assay”, that integrates rheology with optical microscopy for analysing the viscoelastic properties of multicellular spheroids. These spheroids serve as three-dimensional models resembling tissue structures. The innovative technique enables real-time observation and quantification of morphological responses to applied stress using a cost-effective microscope coverslip for constant compression force application. By bridging a knowledge gap in biophysical research, which has predominantly focused on the elastic properties while only minimally exploring the viscoelastic nature in multicellular systems, the i-Rheo-optical assay emerges as an effective tool. It facilitates the measurement of broadband viscoelastic compressional moduli in spheroids, here derived from cancer (PANC-1) and non-tumoral (NIH/3T3) cell lines during compression tests.

This approach plays a crucial role in elucidating the mechanical properties of spheroids and holds potential for identifying biomarkers to discriminate between healthy tissues and their pathological counterparts. Offering comprehensive insights into the biomechanical behaviour of biological systems, i-Rheo-optical assay marks a significant advancement in tissue engineering, cancer research, and therapeutic development.

## Graphical abstract

**Figure:**
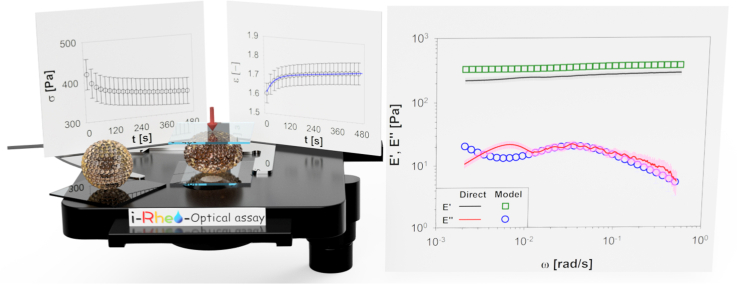

## 1 Introduction

The mechanical characterization of biological specimens, ranging from individual cells to monolayer-cultured cells and complex tissues, has garnered substantial attention in recent scientific literature. This heightened interest is attributed to the broader scientific community's recognition that the mechanical properties of biologically relevant specimens hold the potential to serve as effective biomarkers, facilitating the discrimination between healthy tissues and their pathological counterparts. This discrimination is achieved through the provision of valuable information regarding the frequency-dependent mechanical properties of these specimens, which are intricately regulated by a diverse array of physiological processes encompassing fundamental events such as cell migration [1], wound healing [2,3], and immune responses [[4], [5], [6]]. Moreover, they play pivotal roles in pathological scenarios, with particular emphasis on their involvement in the metastatic progression of cancer [[7], [8], [9]]. The elucidation of the mechanical properties associated with these biological processes may significantly contribute to our understanding of the underlying mechanisms governing health and disease.

Within the field of tissue engineering, the incorporation of multicellular spheroids has arisen as a crucial strategy to bridge the existing knowledge disparity between conventional two-dimensional cell cultures and animal models [[10], [11], [12], [13], [14], [15]]. Multicellular spheroids, indeed, serve as a noteworthy surrogate for animal models, as they faithfully emulate the intricacies of the *in vivo* tissue microenvironment. Consequently, they provide a more accurate representation of biological responses [[16], [17], [18], [19]]. The three-dimensional structures of multicellular spheroids facilitate robust cell-cell interactions, resulting in the establishment of tight junctions that closely resemble those observed in native tissues [10,[20], [21], [22], [23]]. This architectural feature enhances the physiological relevance of multicellular spheroids, contributing to their utility as an intermediate model system that captures aspects of tissue complexity beyond what is achievable in traditional two-dimensional cell cultures.

While the elastic properties of biological systems have garnered substantial attention across various fields, the viscoelastic nature of multicellular systems remains minimally explored in literature [24,25]. Several experimental methodologies have been employed and customized for the investigation of these properties [26], including techniques such as atomic force microscopy (AFM) [[27], [28], [29], [30]], indentation [28], tweezers [31], microfluidics [[32], [33], [34]], micropipettes [35,36], osmotic compression [37], and confocal-rheology [38]. These methodologies are characterized by sophisticated apparatus and necessitate technical skills that may not be readily available.

In this work, we leverage cellular spheroids as a representative model for avascular early-stage tumours, acknowledging their capacity to yield invaluable insights into the nuanced rheological properties of these tumour-like structures. To conduct our study, we subjected spheroids derived from a cancer cell line, PANC-1, to a compression test. The liquid overlay technique was employed for spheroids preparation, selected for its judicious balance of cost-efficiency and ease of management [39,40]. At the core of our rheo-optical compression assay was the utilization of a microscope coverslip to apply a constant load, resulting in the development of a cost-effective and user-friendly technique [41]. Essentially, our innovative approach produces strain-time curves of pivotal significance in elucidating the mechanical properties of the spheroids. Subsequently, data fitting of the resultant deformation was accomplished using a linear viscoelastic model, specifically the Burgers model [42,43], to extract mechanical parameters such as the compressional elastic ($E^{\prime }\left(\omega \right)$) and viscous ($E^{\prime \prime }\left(\omega \right)$) moduli. These parameters contribute to a comprehensive understanding of the rheological behaviour of the investigated cellular spheroids. Moreover, we employed the open-access, model-free executable tool named “i-Rheo” [[44], [45], [46]] to derive the viscoelastic properties of the samples directly from their time-dependent stress and strain curves and conducted comparisons with those obtained from the Burgers model. To corroborate our rheo-optical compression assay, we conducted a similar load test through nanoindentation measurements performed on PANC-1 spheroids and on two biomimetic hydrogels, designed for mimicking biological samples in terms of their mechanical properties [11]. The primary aim of the additional investigation was to further validate the analytical procedure to derive the viscoelastic properties of hydrogels via i-Rheo by means of a comparative assessment with conventional oscillatory bulk-rheology measurements.

Following the successful validation of this methodology, the i-Rheo-optical assay was then applied to a second cell line, NIH/3T3, which served as the non-tumoral control. This was done to establish a robust and efficient tool for the swift assessment of viscoelastic moduli in biological systems across a broad frequency spectrum. This advancement holds significant promise for accelerating therapeutic investigations and gaining deeper insights into the mechanical behaviour of biological materials.

## 2 Materials and methods

### 2.1 Cell culture and spheroid preparation

A fibroblast cell line derived from mouse NIH/Swiss embryos (short: NIH/3T3) and human pancreatic carcinoma cells (short: PANC-1) were cultured at a temperature of 37°C within a humidified environment containing 5% CO_2_. This cultivation was conducted in 2D monolayers using their respective standard growth medium, which was made of Dulbecco's Modified Eagle's Medium (DMEM) supplemented with 10% (v/v) Fetal Bovine Serum (FBS), 1% (v/v) antibiotics (50 units/mL penicillin and 50 mg/mL streptomycin), and 1% (v/v) L-glutamine.

For the preparation of cellular spheroids from both cell lines, the classical liquid overlay technique [40,47,48] was employed. Each well of a 48-well plate (Nunclon Δ Multidishes, flat bottom, ThermoScientific, Nunc 150687) was coated with agarose gel (Ultrapure agarose, Invitrogen, Carlsbad, CA), which facilitates cell collection (at a density of 8∙10^3^ cells/well for NIH/3T3 and 3∙10^3^ cells/well for PANC-1) and the formation of cell-cell adhesions within the meniscus of the non-adhesive concave surface. Subsequently, the 48-well plate was incubated for approximately 7 days to achieve the formation of compact spheroids. For more information on the above process, the reader is referred to our previous publication [49].

### 2.2 Rheo-optical compression assay

The rheo-optical compression test was performed by means of microscopy glass coverslips used as stress loading probe on the cell spheroids sitting within standard 6-well plates. In particular, the cell spheroids were collected from the 48-well plate by using a pipette, and then placed into a 6-well plate containing approximately 3 mL of phosphate-buffered solution (PBS); thus, ensuring no wall effects and a consistent settling of the glass coverslip. For more information on the above process, the reader is referred to our recent publication [41].

During the compression test, images were acquired at a rate of 0.5Hz using an inverted microscope, i.e. an LSM 5 Pascal model by Carl Zeiss Advanced Imaging Microscopy in Jena, Germany. The entire experimental duration was 8 min.

### 2.3 Image analysis

To quantify the impact of mechanical stimuli on spheroid, the morphological changes of the cellular spheroids were assessed in terms of their area (A) and diameter (D) by using an image analysis routine developed with Image Pro Plus 6.0 by Media Cybernetics. The algorithm detected the diametral plane of the cellular spheroids for a total of 240 images for each spheroid of both cell lines.

The stress applied on a spheroid, σ(t), was defined as $\left(F_{w}-F_{A}\right)/A_{t}$, where $F_{w}$ represented the weight of the glass coverslips, $F_{A}$ was the Archimedes force associated with the PBS in which the spheroid was placed, and A_t_ the diametral area of the spheroid measured at the current time. The applied stress was related to the deformation, defined as function strain, ε(t). By assuming constant volume before and after compression, the strain was calculated as $\varepsilon \left(t\right)=h_{t}\left(t\right)/h_{0}={\left(D_{0}/D_{t}\left(t\right)\right)}^{2}$, where D(t) is the diameter of the spheroid as visualized from the compression direction, and h(t) is the spheroid thickness along the direction of compression, i.e. the distance between the two confining glass surfaces. The indices ‘0’ and ‘t’ refer to the function values measured at times zero and current time, respectively. In this work, we approximated the spheroid's volume to that of an oblate spheroid, characterized by three axes a, b, and c, where a = b (=D) > c = h, representing the spheroid's diameter and height, respectively.

### 2.4 Burgers model: from time- to frequency-domain

**Fig. 1:**
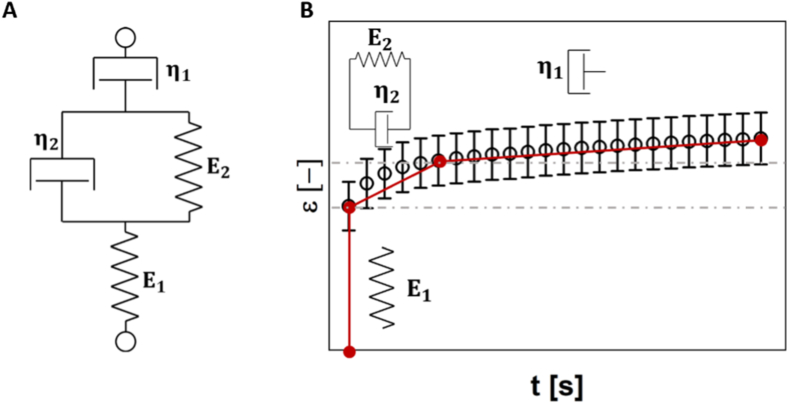
**A)** A schematic diagram of a Burgers material; **B)** A representation of the relative contributions of the individual components of a four-element Burgers model in a creep test, where the strain, ε, is drawn versus time, t.

In the time-domain, in evaluating the viscoelastic properties of cellular spheroids, the correlation between stress (σ(t)) and strain (ε(t)) was subjected to fitting using the Burgers model [50] — a widely recognized model in the biomedical literature for characterizing the linear viscoelastic behaviour of complex materials, ranging from synthetic hydrogel to bone and tissues [42,43,[51], [52], [53], [54], [55], [56], [57]]. Burgers viscoelastic model combines a Maxwell model, *i.e.*, a spring and a dashpot in series, and Voigt model, *i.e.*, a spring in parallel with a dashpot, as schematically shown in Fig. 1.

The one-dimensional differential equation governing this model is expressed as follows:

(1) $$\sigma \left(t\right)+\left(\frac{\eta_{1}}{E_{1}}+\frac{\eta_{2}}{E_{1}}+\frac{\eta_{2}}{E_{2}}\right)\dot{\sigma }\left(t\right)+\frac{\eta_{1}}{E_{1}}\frac{\eta_{2}}{E_{2}}\ddot{\sigma }\left(t\right)=\eta_{2}\dot{\varepsilon }\left(t\right)+\frac{\eta_{1}}{E_{1}}\eta_{2}\ddot{\varepsilon }\left(t\right)$$

Where σ(t) is the axial stress loading, ε(t) is the axial strain response, $\dot{\sigma }\left(t\right)$, $\ddot{\sigma }\left(t\right)$ , $\dot{\varepsilon }\left(t\right)$ , $\ddot{\varepsilon }\left(t\right)$, are their first and second time derivatives, respectively; while $\eta_{i}$ and $E_{i}$ are the viscous and the elastic coefficients of the Newtonian and Hookean elements (with i = 1, 2), respectively.

In the frequency-domain, Equation (1) writes as follows:

(2) $$\hat{\sigma }\left(\omega \right)\left(1+\kappa_{1}i\omega -\tau_{1}\tau_{3}\omega^{2}\right)=\hat{\varepsilon }\left(\omega \right)\left(\eta_{2}i\omega -\tau_{1}\eta_{2}\omega^{2}\right)$$

where, $\hat{\sigma }\left(\omega \right)$ and $\hat{\varepsilon }\left(\omega \right)$ are the Fourier transforms (“ˆ”) of the axial stress σ(t) and strain ε(t), respectively; whereas, $\tau_{1}=\eta_{1}/E_{1}$, $\tau_{2}=\eta_{2}/E_{1}$, $\tau_{3}=\eta_{2}/E_{2}$, $\kappa_{1}=\left(\tau_{1}+\tau_{2}+\tau_{3}\right)$, and i is the imaginary unit ($i^{2}=-1$). Equation (3) provides a means for evaluating the frequency-dependent compressional complex modulus:

(3) $$E^{*}\left(\omega \right)=\frac{\hat{\sigma }\left(\omega \right)}{\hat{\varepsilon }\left(\omega \right)}=\frac{\left(\tau_{2}+\tau_{3}+\tau_{1}^{2}\tau_{3}\omega^{2}\right)}{{\left(1-\tau_{1}\tau_{3}\omega^{2}\right)}^{2}+{\left(\kappa_{1}\omega \right)}^{2}}\eta_{2}\omega^{2}+i\frac{\left[1+\left(\tau_{1}+\tau_{2}\right)\tau_{1}\omega^{2}\right]}{{\left(1-\tau_{1}\tau_{3}\omega^{2}\right)}^{2}+{\left(\kappa_{1}\omega \right)}^{2}}\eta_{2}\omega =E^{\prime }\left(\omega \right)+i\;E^{\prime \prime }\left(\omega \right)$$

which provides information on both the elastic (via the compressional storage modulus $E^{\prime }\left(\omega \right)$) and the viscous (via the compressional loss modulus $E^{\prime \prime }\left(\omega \right)$) nature of the system under study.

In this work, we exploited Equation (3) to evaluate the (non-linear) viscoelastic properties of spheroids either (i) by a regression of the experimental data in the time-domain (i.e., σ(t), ε(t)) by means of Equation (1) to determine the parameters $\eta_{1}$, $\eta_{2}$, $E_{1}$ and $E_{2}$, or (ii) by a direct transformation of the stress & strain raw experimental data by means of i-Rheo [44], which is a model-free algorithm that allows the evaluation of the frequency-dependent complex modulus through the ratio of the Fourier transforms of the axial stress σ(t) and strain ε(t). A direct comparison of the outcomes of the two approaches above is presented hereafter.

### 2.5 Rheological setup

Conventional oscillatory bulk-rheology measurements of the hydrogels were performed by using a stress-controlled rheometer (Anton Paar Physica MCR 302 Instrument) equipped with a parallel plate measuring system of 25 mm diameter (PP25-SN36246). Mechanical properties were investigated by means of strain (γ) sweep tests, spanning from 0.01% to 1% at an angular frequency (ω) of 10 rads^−1^. Measurements were performed on dry hydrogels samples of cylindrical shape, without the addition of water to their edges to avoid swelling. The samples had a diameter of 29.1 mm and an initial thickness of 1.5 mm ± 0.1 mm. The frequency-dependent materials’ linear viscoelastic properties were represented by their shear complex modulus: $G^{*}\left(\omega \right)=G^{\prime }\left(\omega \right)+iG^{\prime \prime }\left(\omega \right)$; which is a complex number whose real and imaginary parts provide information on the elastic and viscous nature of the material under investigation.

### 2.6 Nanoindenter

The effectiveness of the proposed method has been further corroborated by means of measurements performed with a nanoindenter device, referred as Chiaro (Optics11, Netherlands) [58], mounted on top of an inverted phase contrast microscope (Evos XL Core, Thermofisher, UK). All measurements were performed with the same cantilever having a stiffness k = 0.026 N/m and a spherical tip of 46 μm diameter. For each sample, measurements were repeated in at least 3 different spots.

To conduct these measurements, hydrogel samples with a cylindrical shape having a radius of 17 mm and an initial thickness of 1.5 mm ± 0.1 mm were positioned in a petri dish filled with approximately 3 mL of distilled water.

Spheroids were picked using a pipette (preferably with a cut tip to avoid damage during loading) and were seeded in a petri dish previously filled with approximately 3 mL of standard growth medium, few hours before the experiment, allowing the spheroids to adhere to the petri dish but preserving their 3D structure.

The typical creep test was conducted through load control tests to replicate our rheo-optical test conditions. The shear storage and loss moduli at each frequency were calculated for both systems using the i-Rheo method [46,59].

### 2.7 Hydrogels preparation

Hydrogels were synthesized through the crosslinking of polyacrylamide solutions, prepared either with Acrylamide/Bis Solution (40% Acrylamide/Bis Solution, 29:1, Bio-Rad, #1610147) or Acrylamide/Bis Crosslinker powder in a 10:1 ratio (Acrylamide, Bio-Rad, #1610100, and Bis Crosslinker, Bio-Rad, #1610200). The concentrations utilized spanned a range (5%–15% v/v for Acrylamide/Bis Solution and 4%–8% wt for Acrylamide/Bis Crosslinker powder) selected to yield hydrogels exhibiting mechanical properties resembling those found in diverse biological tissues. This systematic variation in concentration allows for the tailoring of hydrogel properties to mimic the mechanical characteristics of a wide variety of tissues [60,61], enhancing their versatility for applications in biomedical and scientific research.

For sample preparation, the requisite amount of polymer and crosslinker was homogenously mixed with distilled water, yielding a final solution volume of 20 mL. Subsequently, 200 μL of ammonium persulfate (APS) with a concentration of 10% w/v was introduced into the solution, initiating the polymerization reaction through the addition of 20 μL of N,N,N′,N'-Tetramethylethylenediamine accelerator (TEMED).

Hydrogel solutions, approximately 10 mL in volume, were cast between two glass plates separated by a spacer measuring 1.5 mm in thickness (Bio-Rad Mini-PROTEAN Spacer Plates with 1.5 mm Integrated Spacers, #1653312). Following polymerization, typically requiring approximately 30 min at room temperature, the glass plates were removed. The resulting gel structures were subsequently molded into cylindrical shapes resembling pills, possessing a thickness of 1.5 mm ± 0.1 mm. This molding process involved the use of a circular cutter to match the diameter of the rheometer tool, ensuring dimensions suitable for subsequent analysis with a nanoindenter.

### 2.8 Statistical analysis

Nanoindentation measurements were repeated at least in 3 different spots for biomimetic hydrogels and biological samples. At least 5 spheroids were analysed for each cell lines (tumoral and non-tumoral) for both nanoindenter and rheo-optical compression tests. Data are expressed as mean ± standard error of mean (SEM).

## 3 Results

Rheo-optical compression tests were initially conducted on PANC-1 spheroids. To validate our technique, a similar creep test was performed by using a nanoindentation test on spheroids and, subsequently, on two biomimetic hydrogels. Concerning the hydrogels, their viscoelastic properties were determined by applying i-Rheo to nanoindentation results, and these outcomes were then compared with those obtained through traditional oscillatory bulk-rheology measurements, as described below. After validating this methodology, we applied the i-Rheo-optical assay to a second cell line, NIH/3T3, which served as the non-tumoral control.

### 3.1 i-Rheo-optical assay PANC-1 spheroids

**Fig. 2:**
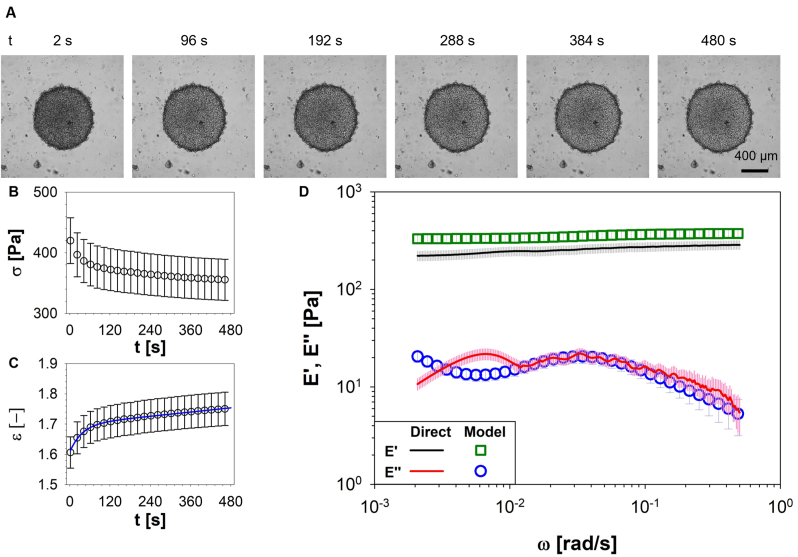
i-Rheo-optical compression assay performed on PANC-1 spheroids. **A)** Representative phase-contrast microscopy images showing the morphological response of PANC-1 spheroids at six different times: 2, 96, 192, 288, 384 and 480 s. Images of the panel are relative to a single spheroid, taken as a qualitative representation of a given experimental condition. Scale bar: 200 μm. **B)** Evolution of stress, σ(t), defined as the ratio between constant loading force and the evolution of spheroid area. **C)** Morphological response of biological system in terms of strain, ε. Experimental data (circle symbols) were fitted by using Burgers model (i.e., Equation (1), solid blue line). **D)** Comparison between the viscoelastic compressional moduli obtained by using either the Burgers model in the frequency domain (i.e. Equation (3), open symbols) or the model-free i-Rheo, applied to the raw data from the compression assay (lines). Data were averaged on at least 10 spheroids. (For interpretation of the references to colour in this figure legend, the reader is referred to the Web version of this article.)

Constant-load creep tests were conducted on PANC-1 spheroids. In Fig. 2A representative phase contrast microscopy images of PANC-1 tumoral spheroids are reported; each image refers to a different time measured from the start of the experiment. Preliminary visual analysis of these images reveals a significant expansion of the spheroids' cross-section perpendicular to the compressional force within the initial seconds of the experiment, swiftly attaining a relatively stable and uniform deformed state. In Fig. 2B, the average value of stress (σ(t)), evaluated across at least 10 spheroids, is plotted as a function of time, t. Due to the constant-load compression, the spheroid's area increased over time, leading to a reduction in σ(t) because of its definition as the ratio between the loading force and the contact area (see **2 section**). The resulting strain history, obtained through image analysis, is shown in Fig. 2C. The number of experimental data points (circles) reported in Fig. 2B and C, has been reduced to improve the readability of the outcomes. Experimental data were fitted by means of the Burgers model (i.e., Equation (1), solid blue line) to determine the coefficients $\eta_{1}$, $\eta_{2}$, $E_{1}$ and $E_{2}$. These coefficients were subsequently utilized to calculate $E^{\prime }\left(\omega \right)$ and $E^{\prime \prime }\left(\omega \right)$ via Equation (3), and the outcomes were compared with those obtained via creep measurements processed by means of i-Rheo, as shown in Fig. 2D, from which a very good agreement between the two methods is apparent.

### 3.2 Cross-methodology validation

#### 3.2.1 i-Rheo-optical assay vs. nanoindentation

**Fig. 3:**
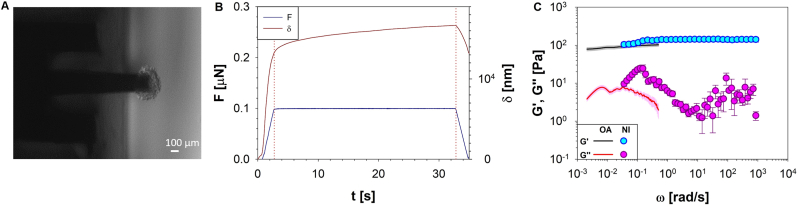
**A)** Representative phase-contrast microscopy images showing the PANC-1 spheroids indented with the cantilever on the left. Image is relative to a single spheroid, taken as a qualitative representation of a specific experimental condition. Scale bar: 100 μm. **B)** The time-dependent relationship between load, F(t), and displacement, δ(t), for to a single spheroid. **C)** Comparison between the viscoelastic shear moduli of the spheroids measured by means of nanoindentation (symbols) and by the i-Rheo-optical compression approach (lines), with the outputs divided by 2.8 to account for the relationship between compressional and shear moduli: E = 2G(1+ν), where ν is the Poisson ratio, assumed to be 0.4 [62] for both the sets of measurements. However, it is important to note that maintaining the assumption of incompressibility, as outlined in Section 2.3 (i.e. ν = 0.5), would result in a decrease in the moduli by approximately 6.6%.

Load control tests were conducted on PANC-1 spheroids using a nanoindenter to establish a comparative analysis with outcomes derived from two distinct methodologies: the rheo-optical compression assay and nanoindentation. In Fig. 3A a representative phase contrast microscopy image of a PANC-1 spheroid is reported. Cellular spheroids were indented at fixed load, using the deflection of the cantilever (on the left in Fig. 3A) as a measure of the displacement. The time-dependent relationship between load, F(t), and displacement, δ(t), showed in Fig. 3B, for each spheroid was assessed in the Fourier domain to quantify its linear viscoelastic properties, as shown in Fig. 3C. The viscoelastic response demonstrated a good agreement between the two methodologies, not only in terms of the absolute values of the moduli but also in their behaviour across the common angular frequency range. This consistency in results between the rheo-optical compression assay and nanoindentation further substantiates the effectiveness of the innovative technique for assessing viscoelastic properties in these biological samples.

#### 3.2.2 Nanoindentation vs. bulk-rheology

**Fig. 4:**
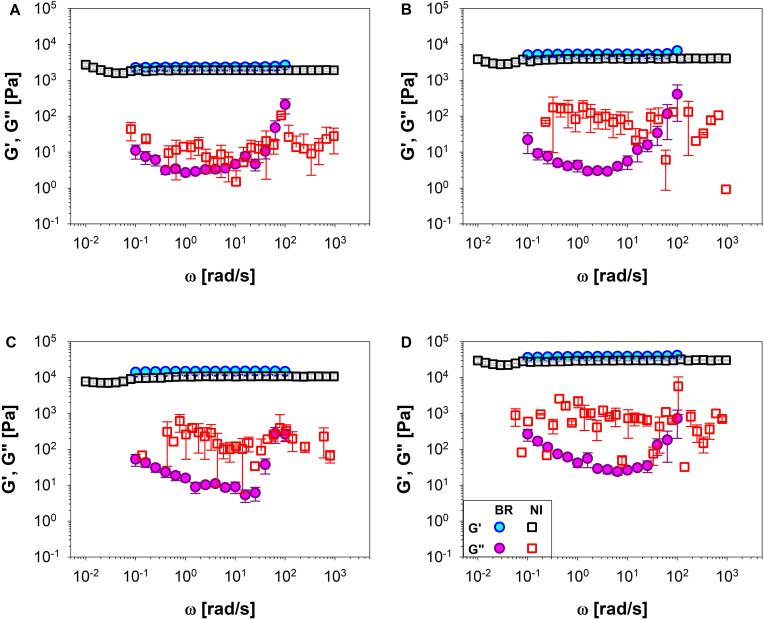
The shear storage modulus, G′, and the shear loss modulus, G″, versus the angular frequency, ω, measured by means of a conventional bulk-rheology rheometer (blue and purple circle symbols) and by means of a nanoindenter (black and red square symbols) for polyacrylamide solution-based hydrogel at different concentrations: 5, 7,10 and 15% v/v (A, B, C, and D, respectively). (For interpretation of the references to colour in this figure legend, the reader is referred to the Web version of this article.)

**Fig. 5:**
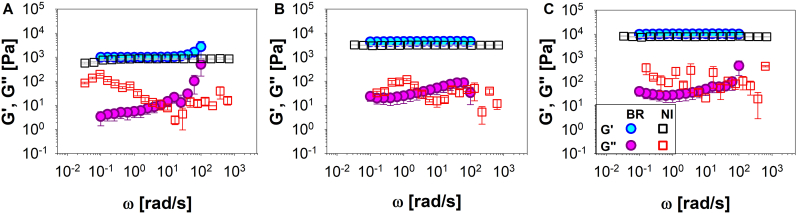
The shear storage modulus, G′, and the shear loss modulus, G″, versus the angular frequency, ω, measured by means of a conventional bulk-rheology rheometer (blue and purple circle symbols) and by means of a nanoindenter (black and red square symbols) for polyacrylamide powder-based hydrogel at different concentrations: 4, 6 and 8% wt (A, B, and C, respectively). (For interpretation of the references to colour in this figure legend, the reader is referred to the Web version of this article.)

To further validate the methodologies presented in this work, load control indentations were also performed on two different polyacrylamide hydrogels, solution- and powder-based. Multiple measurements (≥10) were repeated at different gels concentrations. A comparison between the samples viscoelastic moduli measured by using a conventional bulk-rheology rheometer (filled symbols) and nanoindenter (open symbols) are reported (i) in Fig. 4 for solution-based hydrogels at concentrations of 5, 7, 10 and 15% v/v (**A**, **B**, **C**, and **D**, respectively) and (ii) in Fig. 5 for powder-based hydrogels at concentrations of 4, 6 and 8%wt (**A**, **B** and **C**, respectively). It is important to notice that the agreement between the two methods for G′ values is better than that of G''. This could be related to the fact that the analytical method used to analyse Nanoindentation measurements are based on the Hertz model, which was primarily developed for describing the deformation of purely elastic solid, thus neglecting their viscous behaviour. Additionally, the correlation between σ(t)-ε(t) is approximated to a first-order relation, which does not allow for a more precise evaluation of the Young's modulus.

### 3.3 i-Rheo-optical assay NIH/3T3 spheroids

**Fig. 6:**
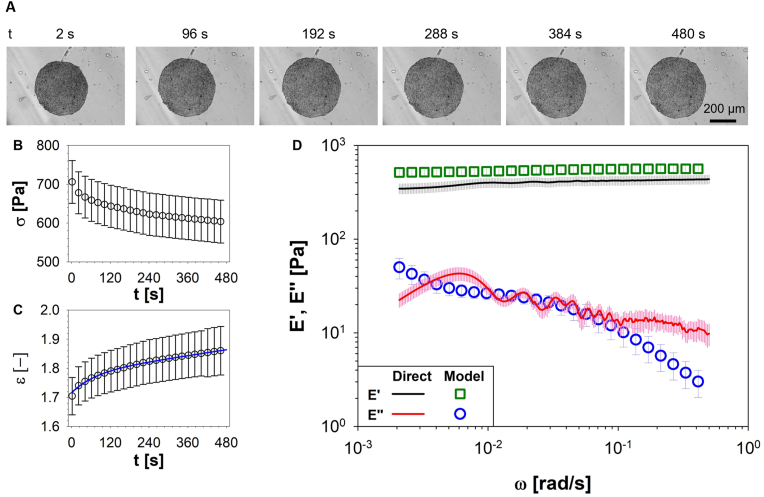
i-Rheo-optical compression assay performed on NIH/3T3 spheroids. **A)** Representative phase-contrast microscopy images showing the morphological response of NIH/3T3 spheroids at six different times: 2, 96, 192, 288, 384 and 480 s. Images of the panel are relative to a single spheroid, taken as a qualitative representation of a given experimental condition. Scale bar: 200 μm. **B)** Evolution of stress, σ(t), defined as the ratio between constant loading force and the evolution of spheroid contact area. **C)** Morphological response of biological system in terms of strain, ε. Experimental data (circle symbols) were fitted by using Burgers model (solid blue line). **D)** Comparison between the viscoelastic compressional moduli of the spheroids obtained either by using Burgers model in the frequency domain (i.e., Equation (3), open symbols) or by means of the model-free i-Rheo algorithm, applied to the raw data from the compression assay (lines). Data were averaged on at least 10 spheroids. (For interpretation of the references to colour in this figure legend, the reader is referred to the Web version of this article.)

For NIH/3T3 cells, illustrative phase contrast microscopy images showing non-tumoral spheroids at different timestamps are presented in Fig. 6A. At a glance, it can be seen that the spheroids primarily expanded during the initial phase of the experiment under the influence of compressive stress. Subsequently, they reach a relatively stable and consistent deformed state. In Fig. 6B and **C**, the average stress values and the corresponding strain history are plotted against time, respectively. Also in this case, the duration of the whole experiment was 8 min, with an image acquisition rate of 0.5Hz. For clarity of the plot, not all the experimental data have been reported in the graph. The fitting parameters $\eta_{1}$, $\eta_{2}$, $E_{1}$ and $E_{2}$ were used to calculate the viscoelastic moduli via Equation (3) and these were compared with those obtained by means of i-Rheo in Fig. 6D. Notably, the results demonstrate a good agreement between the two methods, with a value of $E^{\prime }$ of circa 5.15∙10^2^ Pa and 3.46∙10^2^ Pa from the model and the direct evaluation, respectively, at ω approximately 2∙10^−3^ rad/s. By assuming a Poisson ratio, ν, of 0.4, $G^{\prime }$ is calculated to be 1.83∙10^2^ Pa and 1.23∙10^2^ Pa, respectively. These values are slightly higher than those of single cells from the same cell line (100 ± 10 Pa [63]), potentially attributed to the cell-cell and cell-ECM interactions.

Across the analysed range of frequencies, both PANC-1and NIH/3T3 cell lines exhibited frequency-dependent behaviours in their compressional storage, $E^{\prime }\left(\omega \right)$, and compressional loss, $E^{\prime \prime }\left(\omega \right)$, moduli (refer to Fig. 2D for PANC-1 and Fig. 6D for NIH/3T3). A clear disparity in cellular elasticity is apparent between normal and tumoral cells, with PANC-1 cells being approximately 58.8% softer than normal NIH/3T3 ones, when compared in terms of their compressional elastic modulus $E^{\prime }\left(\omega \right)$; an outcome that aligns well with other findings reported in literature [64]. Similarly, when comparing their viscous components, PANC-1 cells are found to be roughly 50% less viscous than NIH/3T3 spheroids.

**Fig. 7:**
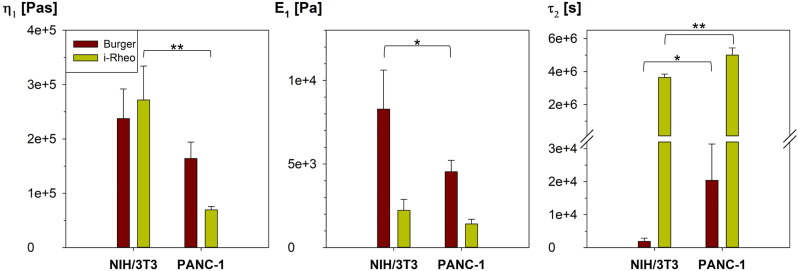
A comparison between the parameters of the Burgers model η_1_, E_1_ and τ_2_, which is defined as the ratio between η_2_ and E_2_, obtained either (i) by fitting Equation (1) to the compression data and then used them to calculate the viscoelastic moduli via Equation (3) for both NIH/3T3 and PANC-1 cell lines (represented in red); or (ii) by fitting the outcomes obtained from the model-free i-Rheo method by using directly Equation (3) for both cell lines (represented in yellow). Statistical significance of the results: *p < 0.05 and **p < 0.005 versus control cells. (For interpretation of the references to colour in this figure legend, the reader is referred to the Web version of this article.)

For a clearer comparison between the i-Rheo and Burger model, data derived from the model-free approach were fitted with Equation (3) to get the same parameter: $\eta_{1}$, $E_{1}$ and $\tau_{2}$, where $\tau_{2}$ is defined as $\eta_{2}/E_{2}$. Both viscous and elastic components were summarized in Fig. 7, for both cell lines using both approaches, with data from the Burger model plotted in red and data from i-Rheo in yellow.

As shown in Fig. 7, the analysis of the parameters of the Burgers model $E_{1}$, $\eta_{1}$ and τ_2_, could serve as possible indicators for discriminating between spheroids based on their viscoelastic properties. In particular, $E_{1}$ represents the instantaneous response, while $\eta_{1}$ is associated with long-term viscous flow. Conversely, $\eta_{2}$ and $E_{2}$ are ‘linked’ to the retarded elastic behaviour, characterizing the middle section of the response curve. According to Fig. 7 and consistent with literature [65], the $\eta_{1}$ value of non-tumoral spheroids (0.267 ± 0.052 MPa∙s) is higher than that of tumoral spheroids (0.164 ± 0.030 MPa∙s). This observation aligns with previous findings suggesting that at a fixed viscosity of the surrounding ECM, a lower viscous cell generates a more pronounced fingering pattern at the interface [26]. Consequently, this parameter may be used to estimate the level of invasion and the metastatic process. Similarly, tumoral spheroids exhibit a lower elastic modulus compared to non-tumoral ones, in agreement with prior research [66]. This observation confirms the critical role of the elastic modulus in understanding the cell's ability to invade blood vessels, which is influenced by their deformability. Ultimately, τ_2_, defined as the ratio between $\eta_{2}$ and $E_{2}$, emerges as the sole parameter among the three investigated capable of distinguishing between the two cell lines for both analytical approaches explored in this paper. Notably, upon comparing the statistical significance of the results shown in Fig. 7, the model-free i-Rheo assay yields a higher level of confidence in distinguishing between the two cell lines.

**Fig. 8:**
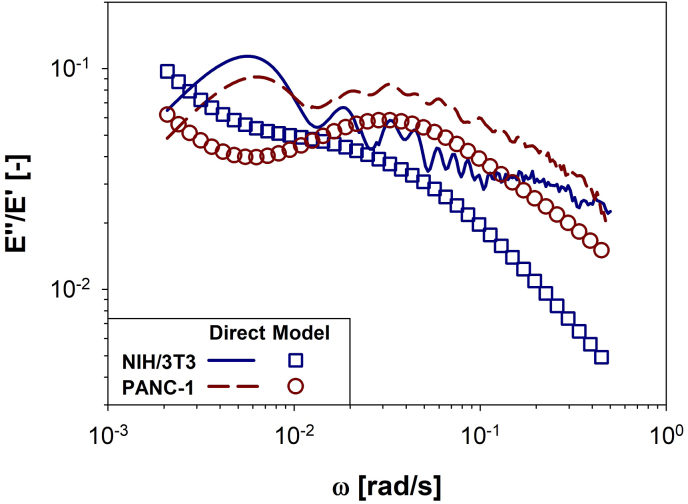
Comparison between the loss tangent, $E^{\prime \prime }\left(\omega \right)/E^{\prime }\left(\omega \right)$, as a function of angular frequency, ω, for both cell lines NIH/3T3 (open squares and continuous line) and PANC-1 (open circles and dashed line). The data have been obtained by using either the Burgers model in the frequency domain (i.e. Equation (3), open symbols) or the model-free i-Rheo, applied to the raw data from the compression assay (lines).

Interestingly, Fig. 8 reveals that in the lower range of the explored frequencies, the non-tumoral NIH/3T3 cells exhibit a higher loss tangent value, $\tan\left(\delta \right)=E^{\prime \prime }\left(\omega \right)/E^{\prime }\left(\omega \right)$, than the cancerous PANC-1 cells, consistently across both i-Rheo-optical assay (lines) and Burgers (symbols) analytical methods. This observation is consistent with the intrinsic characteristics of fibroblasts, whose higher degree of elasticity can be attributed to their distinctive morphology and elevated extracellular matrix (ECM) production in comparison to epithelial cells [67]. In contrast, the cancerous PANC-1 cells exhibit a higher value of tan(δ) at relatively high frequencies (i.e., $\omega >10^{-2}$ rad/s), reflecting their greater deformability when compared to non-tumoral NIH/3T3 cells; an attribute that is congruent with their invasive and migratory capabilities.

## 4 Conclusion

This study introduces a new ‘i-Rheo-optical assay’ that integrates rheology with optical microscopy to analyse the viscoelastic properties of multicellular spheroids. Representing a notable advancement in biophysical research, the assay bridges a critical knowledge gap in understanding the viscoelastic nature of multicellular systems, an area traditionally limited to measuring the elastic modulus. Utilizing a straightforward and cost-effective approach with microscope glass coverslips as the applied load to cell spheroids in standard cell culture plates, this assay was first validated by using PANC-1 tumoral spheroids and subsequently applied to non-tumoral NIH/3T3 spheroids. The strength of our approach lies in its capacity to provide comprehensive viscoelastic moduli data, encompassing both surface cells of spheroids and their interaction with internal spheroid structure and ECM accumulation [68]. This differs from other sophisticated and costly instruments such as AFM or Nanoindenter, which primarily approximate biological samples as purely elastic tissues by using the Hertz model and utilize micrometric tips, thus limiting the investigations to very small regions and neglecting heterogeneities present in both biological samples and hydrogels [69].

By leveraging the open-access, model-free tool i-Rheo, we derived their frequency-depended viscoelastic moduli through Fourier transforms of their time-dependent stress/strain curves, with results aligning well with predictions from the Burgers model. Further validation through (i) nanoindentation tests on PANC-1 spheroids and (i) conventional oscillatory bulk-rheology measurements on biomimetic hydrogels, corroborated the general validity of the novel assay.

The results demonstrate the potential of i-Rheo-optical assay by revealing distinct frequency-dependent viscoelastic moduli values for different cell lines, opening avenues for identifying effective biomarkers critical in discriminating healthy tissues from pathological equivalents. Furthermore, although we assumed a Poisson ratio of 0.4 based on literature, our i-Rheo-optical assay has the capability to educing the correct ν by collapsing E′ and G' [70].

Beyond laboratory applications, the assay's adaptability and scalability make it potentially useful in various biophysical contexts, enriching our understanding of cellular mechanics in diverse biological systems, including biopsies. Moving forward, our future investigations aim to compare different cell types originating from the same tissue, such as breast tissue, with the goal of quantifying variations in elastic and viscous moduli between non-tumoral cells (e.g., MCF-10a) and tumoral ones, while also discerning differences in invasion potential and metastatic capacity (e.g., MCF7 and MBA-MD-231). Additionally, we seek to explore the influence of ECM generated during spheroid formation, as it may impact distinct properties [25] contingent upon the amount of ECM produced and the resulting maturation of spheroids, including the duration of post-preparation incubation. Furthermore, within the realm of biopsies, we aim to quantify the effects of gender (male and female), age, and health status on tissue viscoelastic properties.

This innovation promises significant contributions to tissue engineering, cancer research, and therapeutic development, offering new insights into the study of cellular and tissue mechanics.

## Informed consent statement

Informed consent was waived for this study, as it involved the use of immortalized cell lines obtained from the American Type Culture Collection (ATCC, Manassas, VA).

## CRediT authorship contribution statement

**Rosalia Ferraro:** Writing – review & editing, Writing – original draft, Methodology, Investigation, Formal analysis, Data curation, Conceptualization. **Stefano Guido:** Writing – review & editing, Writing – original draft, Supervision, Project administration, Methodology, Conceptualization. **Sergio Caserta:** Writing – review & editing, Writing – original draft, Supervision, Project administration, Methodology, Data curation, Conceptualization. **Manlio Tassieri:** Writing – review & editing, Writing – original draft, Supervision, Software, Project administration, Methodology, Investigation, Funding acquisition, Formal analysis, Data curation, Conceptualization.

## Declaration of competing interest

The authors declare that they have no known competing financial interests or personal relationships that could have appeared to influence the work reported in this paper.

## Data Availability

Data will be made available on request.

## References

[1] Boot R.C., Koenderink G.H., Boukany P.E. (2021). Spheroid mechanics and implications for cell invasion. Adv. Phys. X.

[2] Vuoso D.C., D'Angelo S., Ferraro R., Caserta S., Guido S., Cammarota M., Porcelli M., Cacciapuoti G. (2020). Annurca apple polyphenol extract promotes mesenchymal-to-epithelial transition and inhibits migration in triple-negative breast cancer cells through ROS/JNK signaling. Sci. Rep..

[3] Ascione F., Caserta S., Guido S. (2017). The wound healing assay revisited: a transport phenomena approach. Chem. Eng. Sci..

[4] Harada Y., Tanaka Y., Terasawa M., Pieczyk M., Habiro K., Katakai T., Hanawa-Suetsugu K., Kukimoto-Niino M., Nishizaki T., Shirouzu M. (2012). DOCK8 is a Cdc42 activator critical for interstitial dendritic cell migration during immune responses. Blood, The Journal of the American Society of Hematology.

[5] Baeyens A.A.L., Schwab S.R. (2020). Finding a way out: S1P signaling and immune cell migration. Annu. Rev. Immunol..

[6] Ding Y., Xu J., Bromberg J.S. (2012). Regulatory T cell migration during an immune response. Trends Immunol..

[7] Gassmann P., Haier J. (2008). The tumor cell–host organ interface in the early onset of metastatic organ colonisation. Clin. Exp. Metastasis.

[8] Mariotto A.B., Etzioni R., Hurlbert M., Penberthy L., Mayer M. (2017). Estimation of the number of women living with metastatic breast cancer in the United States, Cancer Epidemiology. Biomarkers & Prevention.

[9] Ostrom Q.T., Wright C.H., Barnholtz-Sloan J.S. (2018). Brain metastases: epidemiology. Handb. Clin. Neurol..

[10] Hoarau-Véchot J., Rafii A., Touboul C., Pasquier J. (2018). Halfway between 2D and animal models: are 3D cultures the ideal tool to study cancer-microenvironment interactions?. Int. J. Mol. Sci..

[11] Efremov Y.M., Zurina I.M., Presniakova V.S., Kosheleva N.V., Butnaru D.V., Svistunov A.A., Rochev Y.A., Timashev P.S. (2021). Mechanical properties of cell sheets and spheroids: the link between single cells and complex tissues. Biophysical Reviews.

[12] Mehta G., Hsiao A.Y., Ingram M., Luker G.D., Takayama S. (2012). Opportunities and challenges for use of tumor spheroids as models to test drug delivery and efficacy. J. Contr. Release.

[13] Rodrigues T., Kundu B., Silva-Correia J., Kundu S.C., Oliveira J.M., Reis R.L., Correlo V.M. (2018). Emerging tumor spheroids technologies for 3D in vitro cancer modeling. Pharmacol. Therapeut..

[14] Hirschhaeuser F., Menne H., Dittfeld C., West J., Mueller-Klieser W., Kunz-Schughart L.A. (2010). Multicellular tumor spheroids: an underestimated tool is catching up again. J. Biotechnol..

[15] Laschke M.W., Menger M.D. (2017). Spheroids as vascularization units: from angiogenesis research to tissue engineering applications. Biotechnol. Adv..

[16] Costa E.C., Moreira A.F., de Melo-Diogo D., Gaspar V.M., Carvalho M.P., Correia I.J. (2016). 3D tumor spheroids: an overview on the tools and techniques used for their analysis. Biotechnol. Adv..

[17] Chatzinikolaidou M. (2016). Cell spheroids: the new frontiers in in vitro models for cancer drug validation. Drug Discov. Today.

[18] Kim S.J., Kim E.M., Yamamoto M., Park H., Shin H. (2020). Engineering multi-cellular spheroids for tissue engineering and regenerative medicine. Adv. Healthcare Mater..

[19] Nunes A.S., Barros A.S., Costa E.C., Moreira A.F., Correia I.J. (2019). 3D tumor spheroids as in vitro models to mimic in vivo human solid tumors resistance to therapeutic drugs. Biotechnol. Bioeng..

[20] Kapałczyńska M., Kolenda T., Przybyła W., Zajączkowska M., Teresiak A., Filas V., Ibbs M., Bliźniak R., Łuczewski Ł., Lamperska K. (2018). 2D and 3D cell cultures–a comparison of different types of cancer cell cultures. Arch. Med. Sci.: AMS.

[21] Nyga A., Cheema U., Loizidou M. (2011). 3D tumour models: novel in vitro approaches to cancer studies. Journal of cell communication and signaling.

[22] Yamada K.M., Cukierman E. (2007). Modeling tissue morphogenesis and cancer in 3D. Cell.

[23] Horning J.L., Sahoo S.K., Vijayaraghavalu S., Dimitrijevic S., Vasir J.K., Jain T.K., Panda A.K., Labhasetwar V. (2008). 3-D tumor model for in vitro evaluation of anticancer drugs. Mol. Pharm..

[24] Marmottant P., Mgharbel A., Käfer J., Audren B., Rieu J.-P., Vial J.-C., Van Der Sanden B., Marée A.F.M., Graner F., Delanoë-Ayari H. (2009). The role of fluctuations and stress on the effective viscosity of cell aggregates. Proc. Natl. Acad. Sci. USA.

[25] Tsvirkun D., Revilloud J., Giannetti A., Verdier C. (2022). The intriguing role of collagen on the rheology of cancer cell spheroids. J. Biomech..

[26] Elosegui-Artola A. (2021). The extracellular matrix viscoelasticity as a regulator of cell and tissue dynamics. Curr. Opin. Cell Biol..

[27] Andolfi L., Greco S.L.M., Tierno D., Chignola R., Martinelli M., Giolo E., Luppi S., Delfino I., Zanetti M., Battistella A. (2019). Planar AFM macro-probes to study the biomechanical properties of large cells and 3D cell spheroids. Acta Biomater..

[28] Sakuma S., Sato A., Kojima N., Tao F., Arai F. (2017). Force sensor probe using quartz crystal resonator with wide measurement range for mechanical characterization of HepG2 spheroid. Sensor Actuator Phys..

[29] Liu H., Wen J., Xiao Y., Liu J., Hopyan S., Radisic M., Simmons C.A., Sun Y. (2014). In situ mechanical characterization of the cell nucleus by atomic force microscopy. ACS Nano.

[30] Abidine Y., Giannetti A., Revilloud J., Laurent V.M., Verdier C. (2021). Viscoelastic properties in cancer: from cells to spheroids. Cells.

[31] Jaiswal D., Cowley N., Bian Z.C., Zheng G.A., Claffey K.P., Hoshino K. (2017). Stiffness analysis of 3D spheroids using microtweezers. PLoS One.

[32] Ito K., Sakuma S., Kimura M., Takebe T., Kaneko M., Arai F. (2016). Temporal transition of mechanical characteristics of HUVEC/MSC spheroids using a microfluidic chip with force sensor probes. Micromachines.

[33] Hou H.W., Li Q.S., Lee G.Y.H., Kumar A.P., Ong C.N., Lim C.T. (2009). Deformability study of breast cancer cells using microfluidics. Biomed. Microdevices.

[34] Guo Q., McFaul S.M., Ma H. (2011). Deterministic microfluidic ratchet based on the deformation of individual cells. Phys. Rev..

[35] Yanez L.Z., Han J., Behr B.B., Pera R.A.R., Camarillo D.B. (2016). Human oocyte developmental potential is predicted by mechanical properties within hours after fertilization. Nat. Commun..

[36] Blumlein A., Williams N., McManus J.J. (2017). The mechanical properties of individual cell spheroids. Sci. Rep..

[37] Dolega M., Zurlo G., Le Goff M., Greda M., Verdier C., Joanny J.F., Cappello G., Recho P. (2021). Mechanical behavior of multi-cellular spheroids under osmotic compression. J. Mech. Phys. Solid..

[38] Tabatabai A.P., Kaplan D.L., Blair D.L. (2015). Rheology of reconstituted silk fibroin protein gels: the epitome of extreme mechanics. Soft Matter.

[39] Achilli T.-M., Meyer J., Morgan J.R. (2012). Advances in the formation, use and understanding of multi-cellular spheroids. Expet Opin. Biol. Ther..

[40] Costa E.C., de Melo‐Diogo D., Moreira A.F., Carvalho M.P., Correia I.J. (2018). Spheroids formation on non‐adhesive surfaces by liquid overlay technique: considerations and practical approaches. Biotechnol. J..

[41] Ferraro R., Caserta S., Guido S. (2024). A low-cost, user-friendly rheo-optical compression assay to measure mechanical properties of cell spheroids in standard cell culture plates. Advanced Materials Technologies n/a(n/a).

[42] Wintner O., Hirsch‐Attas N., Schlossberg M., Brofman F., Friedman R., Kupervaser M., Kitsberg D., Buxboim A. (2020). A unified linear viscoelastic model of the cell nucleus defines the mechanical contributions of lamins and chromatin. Adv. Sci..

[43] Tong S., Singh N.K., Sknepnek R., Košmrlj A. (2022). Linear viscoelastic properties of the vertex model for epithelial tissues. PLoS Comput. Biol..

[44] Tassieri M., Laurati M., Curtis D.J., Auhl D.W., Coppola S., Scalfati A., Hawkins K., Williams P.R., Cooper J.M. (2016). i-Rheo: measuring the materials' linear viscoelastic properties “in a step”. J. Rheol..

[45] Rivas-Barbosa R., Escobedo-Sánchez M.A., Tassieri M., Laurati M. (2020). i-Rheo: determining the linear viscoelastic moduli of colloidal dispersions from step-stress measurements. Phys. Chem. Chem. Phys..

[46] Chim Y.H., Mason L.M., Rath N., Olson M.F., Tassieri M., Yin H. (2018). A one-step procedure to probe the viscoelastic properties of cells by Atomic Force Microscopy. Sci. Rep..

[47] R. Leek, D.R. Grimes, A.L. Harris, A. McIntyre, Methods: using three-dimensional culture (spheroids) as an in vitro model of tumour hypoxia, Tumor microenvironment, Springer2016, pp. 167-196. 10.1007/978-3-319-26666-4_10. 27325267

[48] Aizel K., Clark A.G., Simon A., Geraldo S., Funfak A., Vargas P., Bibette J., Vignjevic D.M., Bremond N. (2017). A tuneable microfluidic system for long duration chemotaxis experiments in a 3D collagen matrix. Lab Chip.

[49] R. Ferraro, F. Ascione, P. Dogra, V. Cristini, S. Guido, S. Caserta, Diffusion-induced anisotropic cancer invasion: a novel experimental method based on tumour spheroids, AIChE Journal n/a(n/a) e17658. https://doi.org/https://doi.org/10.1002/aic.17678.

[50] Findley W.N., Davis F.A. (2013).

[51] Meng Y., Xia Y., Young T.M., Cai Z., Wang S. (2015). Viscoelasticity of wood cell walls with different moisture content as measured by nanoindentation. RSC Adv..

[52] Chuang S.-F., Lin S.-Y., Wei P.-J., Han C.-F., Lin J.-F., Chang H.-C. (2015). Characterization of the elastic and viscoelastic properties of dentin by a nanoindentation creep test. J. Biomech..

[53] Wang D., Lin L., Fu F. (2021). The difference of creep compliance for wood cell wall CML and secondary S2 layer by nanoindentation. Mech. Time-Dependent Mater..

[54] Shepherd T.N., Zhang J., Ovaert T.C., Roeder R.K., Niebur G.L. (2011). Direct comparison of nanoindentation and macroscopic measurements of bone viscoelasticity. J. Mech. Behav. Biomed. Mater..

[55] Hong X., Stegemann J.P., Deng C.X. (2016). Microscale characterization of the viscoelastic properties of hydrogel biomaterials using dual-mode ultrasound elastography. Biomaterials.

[56] Madani N., Mojra A. (2017). Quantitative diagnosis of breast tumors by characterization of viscoelastic behavior of healthy breast tissue. J. Mech. Behav. Biomed. Mater..

[57] Osika M., Kijanka P. (2024). Ultrasound shear wave propagation modeling in general tissue–like viscoelastic materials. Ultrasound Med. Biol..

[58] Bartolozzi A., Viti F., De Stefano S., Sbrana F., Petecchia L., Gavazzo P., Vassalli M. (2020). Development of label-free biophysical markers in osteogenic maturation. J. Mech. Behav. Biomed. Mater..

[59] Moreno‐Guerra J.A., Romero‐Sánchez I.C., Martinez‐Borquez A., Tassieri M., Stiakakis E., Laurati M. (2019). Model‐free rheo‐AFM probes the viscoelasticity of tunable DNA soft colloids. Small.

[60] S.E. Cross, Y.-S. Jin, J. Rao, J.K. Gimzewski, Nanomechanical analysis of cells from cancer patients, Nano-Enabled Medical Applications, Jenny Stanford Publishing2020, pp. 547-566.

[61] Guimarães C.F., Gasperini L., Marques A.P., Reis R.L. (2020). The stiffness of living tissues and its implications for tissue engineering. Nat. Rev. Mater..

[62] Javanmardi Y., Colin-York H., Szita N., Fritzsche M., Moeendarbary E. (2021). Quantifying cell-generated forces: Poisson's ratio matters. Commun. Phys..

[63] Wottawah F., Schinkinger S., Lincoln B., Ananthakrishnan R., Romeyke M., Guck J., Käs J. (2005). Optical rheology of biological cells. Phys. Rev. Lett..

[64] Kwon S., Yang W., Moon D., Kim K.S. (2020). Comparison of cancer cell elasticity by cell type. J. Cancer.

[65] Rubiano A., Delitto D., Han S., Gerber M., Galitz C., Trevino J., Thomas R.M., Hughes S.J., Simmons C.S. (2018). Viscoelastic properties of human pancreatic tumors and in vitro constructs to mimic mechanical properties. Acta Biomater..

[66] Alibert C., Goud B., Manneville J.B. (2017). Are cancer cells really softer than normal cells?. Biol. Cell..

[67] Qureshi O.S., Bon H., Twomey B., Holdsworth G., Ford K., Bergin M., Huang L., Muzylak M., Healy L.J., Hurdowar V. (2017). An immunofluorescence assay for extracellular matrix components highlights the role of epithelial cells in producing a stable, fibrillar extracellular matrix. Biology open.

[68] Kosheleva N.V., Efremov Y.M., Koteneva P.I., Ilina I.V., Zurina I.M., Bikmulina P.Y., Shpichka A.I., Timashev P.S. (2023). Building a tissue: mesenchymal and epithelial cell spheroids mechanical properties at micro-and nanoscale. Acta Biomater..

[69] Di Lorenzo F., Hellwig J., von Klitzing R., Seiffert S. (2015). Macroscopic and microscopic elasticity of heterogeneous polymer gels. ACS Macro Lett..

[70] Kalcioglu Z.I., Mahmoodian R., Hu Y., Suo Z., Van Vliet K.J. (2012). From macro-to microscale poroelastic characterization of polymeric hydrogels via indentation. Soft Matter.

